# Neonatal aortic coarctation: a spectrum of anatomic lesions repaired trough left thoracotomy in 22 years experience

**DOI:** 10.1186/1749-8090-8-S1-O133

**Published:** 2013-09-11

**Authors:** E Angeli, FD Petridis, G Oppido, L Careddu, R Liberi, L Ragni, R Formigari, M Agulli, G Gargiulo

**Affiliations:** 1 Pediatric and Adult Congenital Cardiac Surgery Unit, S. Orsola-Malpighi Hospital, University of Bologna Medical School, Bologna, Italy; 2 Pediatric and Adult Congenital Cardiology Unit, S. Orsola-Malpighi Hospital, University of Bologna Medical School, Bologna, Italy; 3 Cardiosurgery Intensive Care Unit, S. Orsola-Malpighi Hospital, University of Bologna Medical School, Bologna, Italy

## Background

To define the incidence of aortic recoarctation and intracranial aneurysms (IAs) after neonatal aortic coarctation(CoA) repair through left thoracotomy.

## Methods

One hundred and thirty-four patients underwent CoA repair from January 1990 to December 2012 (mean FU 115.6±82.9 months). Mean age was 11.5±7.6 days (range 0-30 days). Mean weight was 3.0±0.7kg, 26.1% under 2.5kg of weight. 88 patients (65.7%) presented isolated CoA,36 (26.9%) associated VSD, 10 (7.5%) associated complex cardiac defects. All patients were treated through left thoracotomy, 79.9% with end to end extended anastomosis, 7.4% with prosthetic and 12.7% with subclavian patch. Concomitant pulmonary artery banding was performed in 28 patients (21.1%). During follow up all patients underwent thoracic aortic MR and cardiological evaluation. Cerebral MR angiography was performed to avoid the risk of the association between neonatal CoA repair and IAs development.

## Results

No patients with isolated coarctation died. 3 early deaths (2.2%) occurred in patients with complex cardiac defects. Incidence of late mortality was 3.0%. 3/131 died after complex cardiac defects repair, 1/131 for non cardiac reasons. Freedom from balloon angioplasty was 88.1% at 10 years, without statistical difference between patients with body weight <2.5kg and surgical techniques. Severe recoarctation was statistical significant in patients with subaortic valve stenosis (p<0.021). No patients required aortic reinterventions. MR angiography showed no evidence of intracranial aneurysm,or other vascular malformations development.

## Conclusion

In case of neonatal aortic coarctation isolated or associated with cardiac defects requiring concomitant palliative procedures,the left thoracotomy approach appears safe and feasible even in patients with low birth weight without increased risk of aortic recoarctation. The role of potentially associated risk factors for the development of intracranial aneurisms is minimized.

